# COVID-19 pandemic: Impact on the cardiac implantable electronic devices’ implantation rates in Croatia

**DOI:** 10.1371/journal.pone.0284699

**Published:** 2023-04-26

**Authors:** Ivan Zeljkovic, Jerko Ferri, Pavao Mioc, Sandro Brusich, Richard Matasic, Zrinka Jurisic, Janko Szavits Nossan, Lana Maricic, Katica Cvitkusic Lukenda, Vjekoslav Radeljic, Nikola Pavlovic, Sime Manola

**Affiliations:** 1 Department of Cardiology, *Sestre Milosrdnice* University Hospital, Zagreb, Croatia; 2 Department of Cardiology, *Dubrovnik* General Hospital, Dubrovnik, Croatia; 3 Department of Cardiology, *Rijeka* University Hospital, Rijeka, Croatia; 4 Department of Cardiology, *Zagreb* University Hospital, Zagreb, Croatia; 5 Department of Cardiology, *Split* University Hospital, Split, Croatia; 6 Department of Cardiology, Magdalena Clinic, Krapinske Toplice, Croatia; 7 Department of Cardiology, *Osijek* University Hospital, Osijek, Croatia; 8 Department of Cardiology, General Hospital “Dr. Josip Bencevic”, Slavonski Brod, Croatia; 9 Department of Cardiology, *Dubrava* University Hospital, Zagreb, Croatia; Fondazione IRCCS Policlinico San Matteo, ITALY

## Abstract

**Introduction:**

Coronavirus disease 2019 (COVID-19) pandemic has influenced health-care organization worldwide, including management of non-communicable diseases. The aim of this study was to determine the impact of COVID-19 pandemic on cardiac implantable electronic devices’ (CIEDs) implantation rates in Croatia.

**Methods:**

A retrospective, observational, national study was conducted. The data on CIEDs’ implantation rates from 20 Croatian implantation centres, between January 2018 and June 2021, were extracted from the national Health Insurance Fund registry. Implantation rates before and after COVID-19 pandemic started, were compared.

**Results:**

The overall numbers of CIED implantations in Croatia during COVID-19 pandemic were not different in comparison to 2 years pre-COVID-19 time (2618 vs. 2807, p = .081). The pacemaker implantation rates decreased significantly (by 45%) during April (122 vs. 223, p < .001) and May 2020 (135 vs. 244, p = .001), as well as during November 2020 (177 vs. 264, p = .003), but significantly increased during summer months 2020 comparing to 2018 and 2019 (737 vs. 497, p<0.001). The ICD implantation rates decreased significantly by 59% in April 2020 (26 vs. 64, p = .048).

**Conclusion:**

To the authors best knowledge this is a first study including complete national data on CIED implantation rates and COVID-19 pandemic impact. A significant reduction in number of both pacemaker and ICD implants during specific months of the COVID-19 pandemic was determined. However, afterwards compensation in implants resulted in similar total number when the complete year was evaluated.

## Introduction

Coronavirus disease 2019 (COVID-19), caused by the novel SARS-CoV2 virus, started to spread all over the world in December 2019, and has soon confirmed its pandemic potential. The first cases reported in Europe were on 24 January 2020, and by the end of February, more than 1500 daily new cases were reported [1]. The pandemic was officially declared by the World Health Organization on 11 March 2020 [2]. Since the COVID-19 pandemic has started, health care systems around the world began to change in order to provide the optimal care for COVID-19 patients. However, the necessary reorganization of health care systems has largely influenced the management of non-COVID-19 patients, who were underprioritized. Patients with cardiovascular diseases were not an exception to this general trend. Previous publications demonstrated the overall decrease of interventional cardiology procedures during COVID-19 [3–6]. Moreover, a substantial decline in the number of cardiac implantable electronic devices (CIEDs) has also been reported [7–10]. To date, the impact of COVID-19 pandemic on CIEDs implantation rates in Croatia hasn’t been determined, thus, the aim of this study was to explore whether the COVID-19 pandemic has altered the pacemaker and ICD implantation rates in Croatia.

## Methods

This retrospective, observational study considered the total number of CIED implants and its generator replacements in 20 Croatian implantation centres between January 2018 and June 2021. The data were extracted from the Croatian Health Insurance Fund registry, in which the rates of implants and replacements from all centres in Croatia are recorded. Croatian Health Insurance Fund is one obligatory health insurance in Croatia and all the centres are required to report the procedures and the underlying diagnoses for all patients. These data are public and could be accessed on web [11]. The data are published in excel tables that include a detailed information on procedures based on diagnoses. The data presented in this study were completely extracted from these datasets. Out of 20 implanting centres, 12 are implanting both ICDs and pacemakers and 8 pacemakers only (S1 Table). One centre located in Zagreb (capital city) became dedicated COVID-19 hospital, whereas four other city’s hospitals hospitalized only non-COVID-19 patients, and the rest of the hospitals around the country treated both groups of patients.

For the purposes of this study, time has been divided into two periods: "before COVID-19 period" (BCP) and "COVID-19 period" (CovP). In Croatia the first COVID-19 case was diagnosed on February 26^th^, and the COVID-19 epidemic was officially declared on March 16^th^, 2020. Hence, data from March 2020 were excluded from the statistical analysis to avoid over- or under-estimation bias.

The Ethics Committee of the coordinating centre (*Sestre milosrdnice* University Hospital, Zagreb) was consulted waiving the need for approval given the nature of the data.

### Statistical analysis

Continuous variables were presented as means with standard deviations or median with interquartile range in case of skewed distribution. Continuous variables were compared with nonparametric Mann-Whitney U test. Categorical variables were presented as absolute values and/or percentages and were compared using Pearson χ^2^ test. Descriptive statistics were calculated for the available cases. Two-sided P-value of < 0.05 was considered significant. All statistical analyses were performed using SPSS software for Windows, version 22.0 (IBM SPSS Statistics, Armonk, NY, USA).

## Results

The total number of pacemaker implantations during 2020 was lower when compared to an average number of implants in two earlier years, however it did not reach statistical significance (BCP 2807 vs. CovP 2618, p = .081, Table 2). An average of 234 pacemakers per month were implanted in BCP in comparison to 206 pacemakers per month in CovP (p = NS). When the implant numbers were compared for specific months, there was a significant decrease in pacemaker implants during April (by 45%) and May 2020 (by 45%) when compared to both months in 2018/2019 (April 223 vs. 122, p < .001; May 244 vs. 135, p = .001). Also, significant decrease (by 33%) was noted in November 2020 (264 vs. 177, p = .003). In contrast, during summer months (July-August) 2020, there was a significant increase in number of pacemaker implants when compared to 2018/19 (497 vs. 737, p = .001). There was no significant difference between the number of pacemaker implants between the BCP and CovP when the number were compared monthly, bimonthly, quarterly, half-yearly or in other combination (Table 1 and 2, S2 Fig). When we compared first 6 months of 2021 with appropriate months of 2018/2019, there were no significant differences no matter if we compared monthly, bimonthly, quarterly or 6-months data (Tables 1 and 2).

**Table 1:** Impact of COVID-19 pandemic on pacemaker implantation rates in Croatia, single month analysis.

	Pacemaker implantation (n)			p value 2018/2019 vs 2020	p value 2020 vs 2021
Timeline	2018/2019**	2020	2021		
January	186	210	211	.891	.930
February	227	218	223	.845	.757
March	261	249	281	.441	.456
April	223	122	234	**< .001**	. 611
May	244	135	264	**.001**	.444
June	324	228	265	.082	.676
July	251	301	262	.659	.752
August	117	208	216	.222	.890
September	231	286	242	.165	.285
October	257	284	374	.262	.041
November	264	177	119	**.003**	.215
December	239	193	259	.151	.752

* April-May 2018/2019 compared to April-May 2020/2021

** mean value

**Table 2:** Impact of COVID-19 pandemic on pacemaker implantation rates in Croatia, yearly, bimonthly, quarterly and 6-months analysis.

	Pacemaker implantation (n)			p value 2018/2019 vs 2020	p value 2020 vs 2021
Timeline	2018/2019**	2020	2021		
January-December	2807	2618	2926	.081	.752
January-February	447		434	.687	
April-May	467	257	498	.**001**	.473
	467	377		* **.020**	
June-August	497	737	478	**.001**	**< .001**
September-December	958	858	752	.085	.065
November-December	503	370	378	**.013**	.921
April-December	1922	1852	2207	.431	.252
January-June	1374	1454	1395	.910	.931

* April-May 2018/2019 compared to April-May 2020/2021

** mean value

When the number of ICD implants was compared between BCP and CovP, there was a significant decrease of implants during April 2020 by 59% (64 vs. 26, p = 0.048). There was no other statistically significant difference between the BCP and CovP regarding ICD implants, including monthly, bimonthly, quarterly number or any other combination (Tables 3 and 4 and S3 Fig). However, during April and May 2020, 4 out of 12 centres which implant ICD, reported no ICD implants. In addition, there was no significant difference in number of ICD implants during 2021 when compared to 2018/2019 (Table 4).

**Table 3:** Impact of COVID-19 pandemic on ICD implantation rates in Croatia, single month analysis.

	ICD implantation (n)			p value 2018/2019 vs 2020	p value 2020 vs 2021
Timeline	2018/2019**	2020	2021		
January	43	48	38	.992	.878
February	46	64	67	.325	.925
March	55	36	64	.412	.212
April	64	26	51	**.048**	.218
May	53	55	64	.890	.188
June	46	65	49	.562	.596
July	52	63	65	.756	.989
August	37	65	44	.216	.653
September	31	68	62	.121	.898
October	32	64	59	.085	.752
November	57	57	78	.995	.662
December	60	61	68	.937	.802

ICD–implantable cardioverter defibrillator

**mean value

**Table 4:** Impact of COVID-19 pandemic on ICD implantation rates in Croatia yearly, bimonthly, quarterly and 6-months analysis.

	ICD implantation (n)			p value 2018/2019 vs 2020	p value 2020 vs 2021
Timeline	2018/2019**	2020	2021		
January-December	625	673	725	.552	.483
January-February	89	112	124	.452	.821
April-May	118	81	115	.137.	.240
June-August	142	156	109	.091	.432
September-December	226	250	205	.484	.212
November-December	117	118	146	.945	.356
April-December	486	487	536	.992	.742
January-June	306	295	361	.912	.201

ICD–implantable cardioverter defibrillator

**mean value

## Discussion

The main findings of this multi-centre, national, registry-based study, which included data from all 20 CIED implanting centres in Croatia, are the following: 1) the overall numbers of CIED implants during COVID-19 pandemic were not different in comparison to 2 years pre-COVID time; 2) the pacemaker implantation rates decreased significantly during two months after the official declaration of COVID-19 epidemics (April and May 2020), as well as during November 2020; 3) the pacemaker implantation rates significantly increased during summer months 2020 in comparison to 2018/2019; 4) the ICD implantation rates decreased significantly during April 2020.

We analysed the overall national CIED implantation activity in Croatia before and during the COVID-19 pandemic. A significant reduction in a pacemaker implantation rates first two months after the official declaration of COVID-19 epidemic was determined. That was the period of the strictest anti-COVID-19 measures in Croatia. During these periods there was a significant decrease in number of visits in emergency departments that were not related to COVID-19 which we detected as a main reason of the observed decrease in implantation rate. The same trend was observed in November 2020 occurring during the peak of the second “COVID-19 wave” in Croatia which led to the second “lock down” in our country [12]. Monthly data are similar to the results of studies conducted in Italy, Spain, Peru and Portugal [7–10, 13]. Interestingly, in our study we also showed a significant increase in pacemaker implantation rates during summer 2020. This could possibly be explained by the fact that the numbers of COVID-19 cases in Croatia were relatively low, and the hospitals were gradually re-opening for non-urgent visits [12]. Patients who were not electively admitted to the hospital during lockdown, alongside with those who were previously scheduled for implantation, could have contributed to the overall increase in number of reported procedures during this period. Thus, in Croatia the total number of pacemaker implants per year did not differ in the BCP and CovP. There are no previous studies considering complete national data and including impact of different COVID-19 waves on CIED implantation rates [7–10]. Interestingly, there was no difference in implantation rates during 2021 despite having “lockdown” measures and third “COVID-19 wave”. It is important to emphasize that the indications for pacemaker implantations were the same in the pre-COVID period as well as during pandemic.

Similarly, the ICD implantation rates declined continuously, but the statistically significant decrease was noted only in April 2020, whereas this was not noted in the rest of the following months of COVID-19 pandemic, including first 6 months of 2021. However, in one third of Croatian implantation centres in April and May 2020, no ICD implants were reported. Regarding ICD implantation, our data are also in line with previous results collected in Italy, Spain and Peru [7–10]. However, there was no compensation noted during summer period. This could be explained by the healthcare organisation in COVID-19 pandemic, and the fact that one implanting centre in the capital of Croatia (Zagreb) became dedicated COVID-19 centre, and the remaining 4 centres managed only non-COVID-19 patients. Determination of casual connections is beyond the scope of this paper, but the results provide a ground for a discussion on potential causes that have led to the observed changes. Arbelo et al. [8] and Boriani et al. [7] in their papers speculated on different moments that are likely to give rise to the reduction in implantation rates in Spain and Peru, respectively. Government directives on cancelling the elective procedures have formally made the treatment of non-urgent patients impossible. What is more, fear of contagion, associated with the individual perception of the risk present in the hospital areas could be a second important cause. Finally, during COVID-19 “lockdown” the collective awareness of non-COVID-19 pathologies was reduced. Taking into consideration that the SARS-CoV2 has become almost ubiquitous and the fight against the pandemic is similar worldwide, we think that the underlying causes of the reductions in implant rates are similar, particularly comparing the countries within the European Union. Lockdown during COVID-19 pandemic caused reduction in emergency departments visits not only in Croatia, but also in other European countries [14], but on the other hand SARS-CoV2 infection and its complications, alongside with medication used in treatment COVID-19 patients contributed to the relatively constant CIEDs’ implantation rates during pandemic. Transient decrease in implantation rates was due to lockdown, however in the following months the numbers were increasing. We see it as a “compensation” period. During summer 2020 there was not statistically significant increase in number of CIEDs implantation, but in July 2020 there was the greatest number of implanted pacemakers in last two years (301; Table 1, S2 Fig). We argue that the reason for the observed change in trends could be the fact that only highly symptomatic patients visited emergency departments during lockdown, as discussed by Colivicchi et al. and Baldi et al. [14, 15], and the others were just postponing their visits to the period after the lockdown.

This multicentre retrospective study has several limitations. First, due to the limitation in registry’s data, especially during the COVID-19 pandemic, we have not been able to extract detailed data on relevant variables such as patients’ socio-demographic status, indication for CIED implantation, device type or clinical presentation. Second, the observational nature of the study did not allow any conclusion to be drawn in relation to the underlying causes for the significant variations in CIED implantation rates during the COVID-19 pandemic.

## Conclusion

A significant reduction in total number of pacemaker implants during April, May and November 2020 was determined in Croatia during the COVID-19 pandemic, whereas an increase of pacemaker implants was observed in the period between two “COVID-19 waves”. Regarding the ICD implants, significant decrease was noted only in April 2020, with no significant, but gradual compensation afterwards. Further studies are needed to elucidate the underlying causes of these trends.

## Supporting information

S1 Table Cardiac implantable electronic device implantation centres in Croatia. (PDF)

S1 Fig Total numbers of CIEDs implantations. (DOCX)

S2 Fig Pacemaker implantations monthly. (DOCX)

S3 Fig ICD implantations monthly. (DOCX)
