# Supplementary material for: COVID-19 pandemic: Impact on the cardiac implantable electronic devices’ implantation rates in Croatia
Source: PLoS One. 2023 Apr 26;18(4):e0284699. doi: 10.1371/journal.pone.0284699 (PMC10132659; doi:10.1371/journal.pone.0284699)
Supplement: S1 Table — (PDF) [file pone.0284699.s001.pdf]

Supplemental Table 1 Cardiac implantable electronic device implantation centres in Croatia

**Both ICD and pacemaker implantation centres in Croatia**
Dubrava University hospital
Dubrovnik General hospital
Magdalena Clinic
Merkur University hospital
Osijek University hospital
Rijeka University hospital
Sestre milosrdnice University hospital
Slavonski Brod General hospital
Split University hospital
Sveti Duh University hospital
Zadar General hospital
Zagreb University hospital
**Pacemaker implantation centres in Croatia**
Čakovec General hospital
Karlovac General hospital
Koprivnica General hospital
Našice General hospital
Požega General hospital
Pula General hospital
Thalassotherapia Opatija Specialized hospital
Varaždin General hospital

ICD – implantable cardioverter defibrillator.
