# Supplementary material for: COVID-19 pandemic: Impact on the cardiac implantable electronic devices’ implantation rates in Croatia
Source: PLoS One. 2023 Apr 26;18(4):e0284699. doi: 10.1371/journal.pone.0284699 (PMC10132659; doi:10.1371/journal.pone.0284699)
Supplement: S1 Fig — (DOCX) [file pone.0284699.s002.docx]

**S1 Fig. Total numbers of CIEDs implantations.**
